# Perspectives on health and well-being in social sciences

**DOI:** 10.3402/qhw.v11.31468

**Published:** 2016-05-09

**Authors:** Carin Nyman, Åke Nilsén

**Affiliations:** School of Health and Welfare, Halmstad University, Halmstad, Sweden

From a social scientific perspective, the field of health and well-being is differentiated with contributions of a wide range of topics, methodological approaches, and interdisciplinary research. This may challenge the understanding of health and well-being but can also make an important contribution to the development of the field. This thematic cluster presents new qualitative research on the importance of supportive structures addressing the societal and the organizational level in order to promote health and well-being in the individual. Social sciences have a double identity, being a science contributing with research on society and interacting processes between different levels in the society, and an identity taking on a critical assignment. The cluster addresses this double identity through research aiming to promote sustainable well-being. As guest editors of this thematic cluster in the *International Journal of Qualitative Studies on Health and Well-being*, we would like to present four studies describing different angles of individual experiences and views of the need of supportive structures. Highlighted areas are health promotion activities and shared decision-making processes in mental health care services for individuals suffering from mental ill-health. Furthermore, supportive structures in relation to experiences in professionals and health promotion interventions in work places are discussed. Contemporary societies meet different challenges concerning health and well-being. The overarching objective of this thematic cluster is to increase the knowledge that influencing factors for improved well-being in individuals often are to be found at societal and organizational level. To promote health and well-being in individuals, societal and organizational systems and actors need to interact with the individual as signified by the contribution of research in this thematic cluster. Moreover, the thematic cluster gives an understanding of the variety of approaches in qualitative studies on health and well-being within social sciences.

Living and working conditions are strong determinants of individuals’ physical, mental, and social health and well-being (Marmot, Allen, Bell, Bloomer, & Goldblatt, 2012). Applying a social science perspective on health and well-being means the recognition of the importance of influencing structures and pathways on a societal, organizational, and individual level. Moreover, supportive structures for individuals’ health and well-being are to a great extent shaped by the interactive process between these levels where social context, social position, and resources play an important role (Diderichsen, Evans, & Whitehead, 2001). Contemporary societies are complex and dynamic and require a reflexive understanding and methodology when aiming at increasing the knowledge of supportive structures for health and well-being. The social sciences have a double identity in the sense of being both a science contributing to research *on* society conducted with scientific methods based on an epistemological realism, *and* an identity taking on a critical assignment (Habermas, 1988). This critical assignment is a reflective approach in relation to various social phenomena in the society as well as towards the scientific activity in itself. The social sciences then are practiced based on a traditional methodology shared with other academic subjects, as well as a reflexive methodology based on a constructivist epistemology. The reflexive methodology comes from the very nature of the research object: the social aspects of human actions. When transferred to the field of health and well-being, this double identity can be understood as research *on* health and well-being and research *about* health and well-being. The research *on* health and well-being from a social scientific perspective would then level with other research traditions in the sense that the object of research constitutes different social aspects of health and well-being. The research *about* health and well-being from a social scientific perspective would focus on how the field is formed, what issues are at stake, and how this is done from a critical perspective applying a reflexive methodology (Alvesson & Sköldberg, 2009). In this thematic cluster, however, there is domination of traditional methodology in the contributions, with a common denominator of qualitative studies, but differing in approaches.

Studies have shown that mental ill-health problems are on the rise in both young people and among people in working age in Sweden (Danielsson & Berlin, 2012; Sandmark & Renstig, 2010). This raises an emerging need for health promotion initiatives where different actors on societal and organizational level work together with the individual in the development of supportive structures. In this thematic cluster, the included studies by Grim, Rosenberg, Svedberg, and Schön (2016) and by Jormfeldt and Hallén (2016) contribute to valuable understanding of the importance of supportive structures for individuals suffering from mental illnesses. In the work by Jormfeldt and Hallén, the discourse of mental illness is highlighted through written narratives by service users’ diagnosed with schizophrenia and their relatives’ concerning experiences of support in everyday life. Despite the knowledge that individuals diagnosed with schizophrenia often are at risk for an exacerbated well-being, the responsibility for everyday health promotion activities between authorities are not defined. The findings show a lack of support in everyday life, for example, in facilitating the practice of a healthy life style (Jormfeldt & Hallén, 2016). The findings may raise incitements for the societal level, that is, local authorities, to further develop health-promotive support structures and activities targeting the everyday needs of individuals with mental ill-health. Another example of a supportive structure within the field of mental health is shared decision-making which is investigated in the study by Grim et al. As in the study by Jormfeldt and Hallén, individuals with own experience of psychiatric illness and use of mental health care services are at focus. With an inductive and a deductive approach using focus group interviews, a thorough understanding is developed revealing the importance of informational as well as decisional needs among users of mental health services. This study makes a contribution to the understanding of what are the important constituents in shared decision-making among individuals with mental health problems. In addition, attitudinal, cognitive, and relation-based factors are discussed in order to reduce potential barriers for individuals to get involved in shared decision-making models and processes (Grim et al., 2016). Using different qualitative methods, these studies highlight the importance of participatory approaches where individuals’ experiences and views can form the knowledge base for health professionals and policy makers in the work for supportive structures. In an earlier study in *International Journal of Qualitative Studies on Health and Well-being*, another example of supportive structures as a response to a new welfare context was described among young people with intellectual disabilities (Tideman & Svensson, 2015).

Furthermore, Cuesta and Rämgård and Stenberg show that qualitative methodology is well suited for research on an organizational level in different work place settings. Cuesta and Rämgård aim at examining the use of an intersectional perspective as a way of increasing the knowledge on work place–based power structures. With focus group interviews, they focus at staff experiences and reflections from their work in an elderly care home for foreign-born persons with dementia. The need for an intersectional perspective that put a multifaceted focus on power relations is discussed as a tool for a “consciousness-rising” process which positively can affect well-being among professionals (Cuesta & Rämgård, 2016). The work by Stenberg (2016) gives another example of research where perspectives on health and well-being can be discussed from an individual and from an organizational and societal level. Narratives on fine artists staging artistic projects in different workplace settings are analysed and the meaning and importance of culture activities as a creative and health-promoting resource in modern working life is analysed. A two-folded result emerges showing that such artistic projects can be an important actor for supporting health and well-being at workplaces not at least by supporting social interaction. However, this study also indicates that this contribution can hamper the opportunity to actually perform and develop as an artist and thus entail adverse health developments and negatively affect the professional's own well-being (Stenberg, 2016). From different settings, the studies give an understanding of how the organizational level can be related to individuals’ construction of subjectivity in their professional roles, which in turn can affect their health and well-being. The study by Stenberg furthermore pinpoints a professional group whose working situation seldom is highlighted in occupational studies on health and well-being.

Societies today face emerging challenges concerning health and well-being. Especially the increase in mental ill-health among young people and people in working age is worrying. This call for attention and action and a need for interdisciplinary approaches have been advocated. The results from the included studies show that research from a social scientific perspective can make an important contribution to the knowledge development by including influential processes also originating from a societal and an organizational level. Finally, this thematic cluster will give an understanding of the variety of approaches within the social sciences and by this make an inspiring contribution for future submissions to the *International Journal of Qualitative Studies on Health and Well-being* of social science research.

*Carin Nyman*, PhD School of Health and Welfare, Halmstad University Halmstad, Sweden Email: carin.staland_nyman@hh.se*Åke Nilsén*, PhD School of Health and Welfare, Halmstad University Halmstad, Sweden
